# Treatment Engagement in Specific Psychological Treatment vs. Treatment as Usual for Adolescents With Self-Harm: Systematic Review and Meta-Analysis

**DOI:** 10.3389/fpsyg.2019.00104

**Published:** 2019-02-04

**Authors:** Sze Ngar Vanessa Yuan, Ka Ho Robin Kwok, Dennis Ougrin

**Affiliations:** Child and Adolescent Psychiatry, King's College London, London, United Kingdom

**Keywords:** self harm, randomized controlled trials, meta-analysis, self injurious behavior, psychotherapy

## Abstract

**Background:** Self-harm is a major public health problem. It is one of the best predictors of suicide in adolescents. Despite recent advances in the understanding of self-harm, poor treatment engagement remains a significant clinical obstacle.

**Objectives:** The purpose of this meta-analysis is to update and extend previous research investigating treatment engagement with specific psychological treatments (SPT) vs. treatment as usual (TAU) in adolescents who self-harm.

**Methods:** Data sources were identified by searching the Medline, PsychINFO, EMBASE, and PubMed databases as of October 2017. Randomized Controlled Trials (RCTs) comparing SPT and TAU in adolescents (through age 18 years) with self-harm were included.

**Results:** The results show that 12 RCTs investigating 1,255 young people were included in the meta-analysis. The proportion of adolescents not completing four or more sessions in SPT was significantly lower (28.4%, 179/630) than TAU (45.9%, 287/625), RR = 0.64 (95% CI:0.51 −0.79), *p* < 0.0001. There were significantly more adolescents engaged with SPT than TAU.

**Conclusions:** Specific psychological treatments should be offered to adolescents with self-harm to maximize treatment engagement. Engaging adolescents with psychological treatment is necessary although not sufficient to achieve treatment goals.

## Introduction

Suicide is the second or third leading cause of death in adolescents in Western countries and a major cause of death in developing countries (Hawton et al., 2012; World Health Organization, 2014). Self-harm is also a strong predictor of death by suicide in adolescents and a major public health concern in many countries. Thirteen to Forty-five percentage of adolescents have engaged in self-harm at some point of their lives in community samples, while this rate is up to 40–60% in clinical samples (Nock, 2010). Self-harm in adolescents is associated with 50- to 100-fold increase in the risk of death by suicide. It is also associated with a wide range of psychiatric disorders, such as depression and borderline personality disorder, and high health economic expenditure (National Institute for Health Care Excellence, 2011). Given the high rates of self-harm in adolescents, a recently developed screening too, the Self Harm Questionnaire (SHQ) has improved on identification and prediction of self-harm (Ougrin and Boege, 2013). Twenty percentage of those who disclosed self-harm on the SHQ did not have self-harm recorded in their clinical records and it was later found that self-harm had occurred. The availability of such screening tool helps to identify adolescents who are at an increased risk for suicide. A combination of clinical assessment and self-report questionnaire would be optimal for identification of self-harm in adolescents.

Several psychological therapies have shown an impact on self-harm ideation and behavior in adolescents (Ougrin et al., 2015). However, a systematic review revealed that only over half of these programs had a significant effect on self-harm, suicidal ideation, or suicide attempts (Calear et al., 2016). On the other side, poor attendance and engagement remains to be a significant obstacle in delivering these interventions (Fortune and Hawton, 2005). Previous research indicates that community treatment is poorly attended, with 25–50% of self-harming adolescents reported not to attend any follow-up sessions (Taylor and Stansfeld, 1984; Granboulan et al., 2001). Fifty to Seventy-Seven percentage of adolescents disengage from treatment (Trautman et al., 1993; Haw et al., 2002; Groholt and Ekeberg, 2009), while around 50% of adolescents attend four or fewer outpatient follow-up sessions (Spirito et al., 1992; Groholt and Ekeberg, 2009). Disengagement is a problematic coping style incorporating problem avoidance, wishful thinking, social withdrawal, and avoidance of negative emotions and could lead to poor psychosocial outcome (Votta and Manion, 2004).

There has been an increasing number of controlled studies of specific psychological therapy for adolescents who self-harm. A meta-analysis was conducted in 2011 to investigate whether specific psychological therapies vs. usual care increase engagement in adolescents who self-harm, but no significant difference was found (Ougrin and Latif, 2011). The small number of studies did not allow for further analysis such as evaluating the moderators. Another meta-analysis investigated the effects of specific therapeutic treatment and intervention in reducing suicidal and non-suicidal self-harm in adolescents (Ougrin et al., 2015). Overall, specific pharmacological, social or psychological therapeutic interventions were more effective than usual care including treatment as usual, enhanced treatment as usual, supportive relationship treatment and hospitalization. There is also evidence that intensive community treatment is associated with reduced risk of multiple self-harm in comparison with standard inpatient treatment (Ougrin et al., 2014, 2018; Kwok et al., 2016). Treatment engagement is essential for treatments and interventions to be effective. Therefore, this meta-analysis seeks to update and extend previous research in comparing treatment engagement between psychological therapy and treatment as usual. The availability of newer research studies allows for stricter inclusion criteria and further analyses including moderator analyses. This provides more generalizable findings, leading to greater insight to future research and clinical work.

## Methods

### Inclusion Criteria

We included all randomized controlled trials of specific psychological treatment (SPT) compared to treatment as usual (TAU) for adolescents through age 18 who have self-harmed at least once. Adolescents from different cultural background and socioeconomic statuses are considered. SPT is defined as any theoretically coherent non-pharmacological intervention that are manualized or replicable by others. Interventions considered including home-based intervention, group psychotherapy, family therapy, and therapy focused on the adolescent, etc. TAU is defined as any intervention that reflects the usual care in a given treatment setting with patients receiving typical follow-up appointments and services.

### Exclusion Criteria

We excluded studies in which adolescents who self-harm, parasuicidal behaviors, suicidal ideation, or behavior was not presented as a main inclusion criterion; studies that involved pharmacological intervention; studies with interventions that did not require young people to attend treatment sessions; and studies that did not measure engagement systematically, such as recording the number of attended sessions of each participant.

### Identification and Selection of Data

Articles were identified by systematically searching PsycINFO, PubMed, Embase and OVIDMedline databases to October 2017. The MeSH terms used were “self injurious behavior,” “suicide, attempted,” “self mutilation,” “suicide,” “overdose,” and “self harm, deliberate.” Limits of age group (0–18 years old) and of publication types (randomized controlled trials) were applied. The search results were imported into EndNote (version X7) and all duplicates were removed.

The reference lists and cited articles were searched and relevant studies were evaluated for inclusion. Key investigators from the United Kingdom, United States, Australia and Norway were contacted for any unpublished studies or to clarify details of the published studies.

The search was completed by two of the authors (SY and KK) independently. The two authors screened the titles, abstracts, and full text articles to determine the eligibility of the studies. There were no disagreements during consensus meeting.

Allocation concealment was used as a proxy to assess the methodological quality of the studies. Allocation concealment is a procedure for protecting the randomization process so that the treatment to be allocated is not known until the participant is in the study. Allocation concealment was rated using the following quality ratings: 1 = adequate concealment (e.g., sealed envelope), 2 = unclear concealment, and 3 = inadequate concealment (e.g., open random number tables). Jadad score was also calculated for each included studies (Jadad et al., 1996). Jadad score is an indicator of methodological quality, which assesses the quality of randomization, blinding procedures, and description of withdrawals and dropouts. The score ranges from 0 to 5 while studies scoring 3 or above would be considered as good quality.

Self-harm was defined as an act with a non-fatal outcome in which an individual deliberately initiated behavior intended to cause self-injury, ingested a substance in excess of prescribed or generally recognized therapeutic dose, ingested recreational or illicit drug that the person regarded as self-harmful, or ingested a non-ingestible substance or object (Hawton et al., 2002). Engagement was defined as attending four or more psychotherapeutic treatment sessions, in line with previous literature (Wood et al., 2001; Spirito et al., 2002). We contacted key investigators for clarifications wherever needed.

### Statistical Analysis

We used attending four or more psychotherapeutic treatment sessions to calculate the risk ratio. We dichotomized the subjects into two different groups by using attending four or more treatment sessions as a cutoff. Data were obtained by contacting key investigators if they were not already specified in the paper. RevMan (Version 5.2), a computer program designed to support Cochrane reviews and meta-analyses, was used to calculate the pooled effect size. Each study was weighted in proportion to its sample size and tau^2^ (the estimated variance of the true effect sizes).

There was moderate heterogeneity as indicated by the I^2^ statistic. I^2^ describes the percentage of total variation between studies that is due to heterogeneity rather than by chance (Higgins et al., 2003). In order to allow for heterogeneity, mean risk ratio was calculated with random effects model (DerSimonian and Laird, 1986). A random effects model assumes that individual studies are estimating different treatment effects due to the diversity of methodology and clinical interventions. A funnel plot was utilized to assess the presence of publication bias for the main hypothesis of treatment engagement in SPT vs. TAU for adolescents with self-harm. Egger's test was used to formally assess publication bias (Egger et al., 1997). After the removal of the studies with Jadad ≤2 in the sensitivity analysis, there is little variation between the studies, making a fixed effects model more appropriate.

Finally, meta-regression was completed to assess the influence of number of training sessions (single vs. multiple), year of study, mean age (years), and gender percentage on the effect size.

## Results

### Included Studies

**Figure 1:**
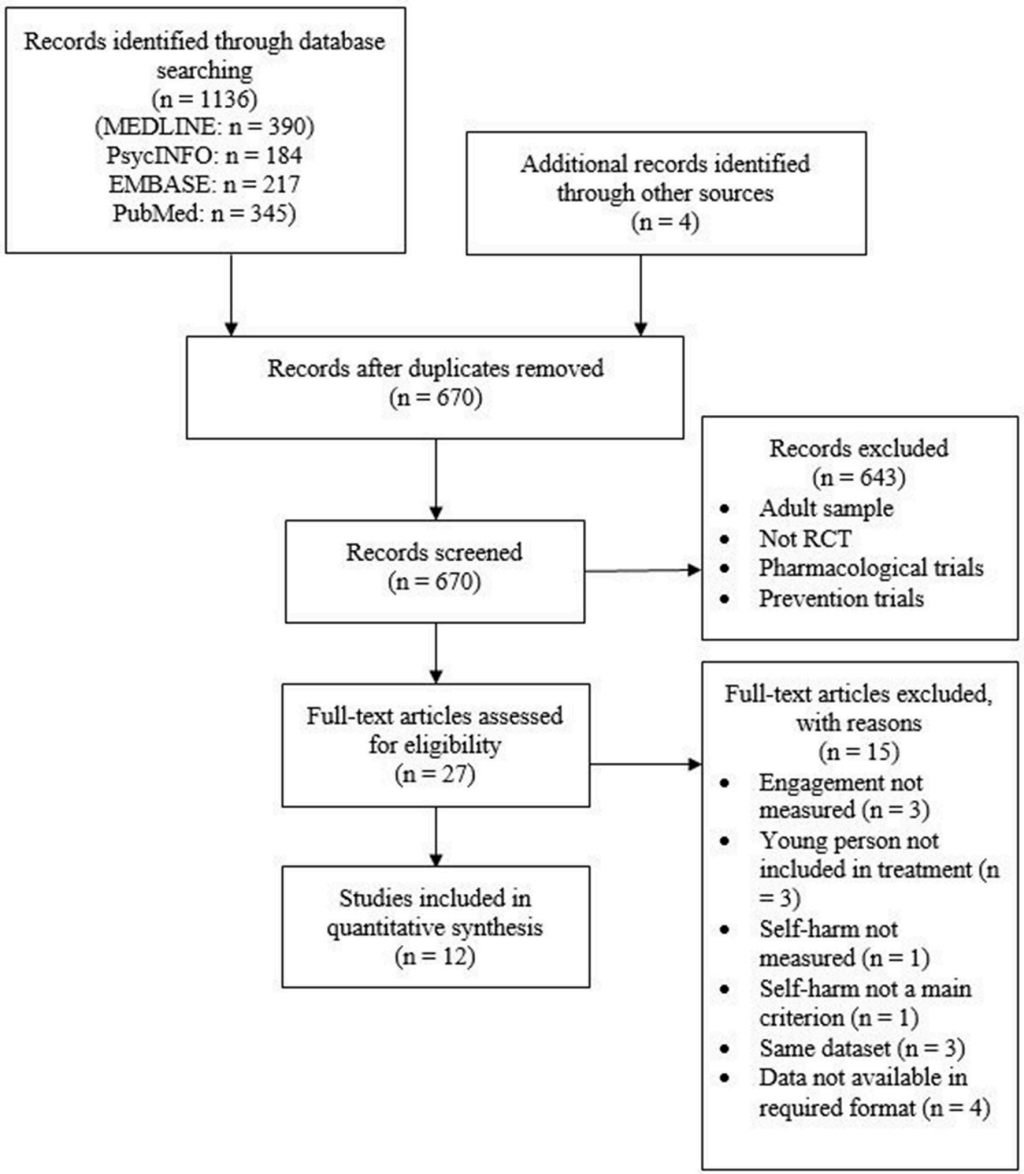
Flow of studies.

The original search resulted in 1,136 articles and 470 duplicates were removed. Four additional articles were identified through the reference lists and from the sharing of other researchers. The remaining articles were screened for abstract and 27 articles were examined for full-text (Cotgrove et al., 1995; Harrington et al., 1998; Wood et al., 2001; Spirito et al., 2002; Huey et al., 2004; Donaldson et al., 2005; King et al., 2006, 2009; Chanen et al., 2008; Hazell et al., 2009; Schuppert et al., 2009, 2012; Diamond et al., 2010; Asarnow et al., 2011, 2017; Esposito-Smythers et al., 2011; Green et al., 2011; Ougrin et al., 2011, 2013; Rossouw and Fonagy, 2012; Alavi et al., 2013; Hughes and Asarnow, 2013; Pineda and Dadds, 2013; Mehlum et al., 2014, 2016; Goodyer et al., 2017; Wharff et al., 2017). A summary of the process is presented in Figure 1. A total of 16 articles met full inclusion criteria as randomized controlled trials of adolescents with self-harm of suicidality as primary presenting problem (Harrington et al., 1998; Wood et al., 2001; Spirito et al., 2002; Donaldson et al., 2005; Chanen et al., 2008; Hazell et al., 2009; Schuppert et al., 2009, 2012; Diamond et al., 2010; Asarnow et al., 2011; Esposito-Smythers et al., 2011; Green et al., 2011; Ougrin et al., 2011; Rossouw and Fonagy, 2012; Mehlum et al., 2014; Wharff et al., 2017). Four of the studies did not report the data in the format required and hence were not included in the meta-analysis (Schuppert et al., 2009, 2012; Rossouw and Fonagy, 2012; Wharff et al., 2017). The characteristics of the included 12 studies are presented in Table 1.

**Table 1:** Characteristic of studies.

**First author, year, country**	**N**	**Age**	**Female %**	**Inclusion criteria**	**Interventions**	**Control**	**ITT**	**Allocation**	**Follow-up**
Harrington et al. (1998), UK	162	10–16	89.5	Diagnosis of deliberate self-poisoning	Home-based family intervention + TAU	TAU	Subjects randomized	Concealed	6 mos.
Wood et al. (2001), UK	63	12–16	77.8	Self-harm repeater in outpatient service	Developmental group psychotherapy + TAU	TAU	Subjects randomized	Concealed	7 mos.
Spirito et al. (2002), US	76	12–18	90.5	Suicide attempters receiving care in ED or pediatric ward	Compliance enhancement Intervention + Standard disposition planning	Standard disposition planning	Subjects completed	Not specified	3 mos.
Donaldson et al. (2005), US	39	12–17	82.1	Suicide attempters in ED or inpatient unit in child psychiatric hospital	Skills-based treatment (SBT)	Supportive relationship treatment (SRT)	Subjects started	Not specified	6 mos.
Chanen et al. (2008). AU	86	15–18	75.6	DSM-IV criteria for BPD	Cognitive analytic therapy (CAT)	Good clinical care (GCC)	Subjects randomized	Concealed	24 mos.
Hazell et al. (2009), AU	72	12–16	90.3	Self-harm repeater in outpatient service	Developmental group psychotherapy	TAU	Subjects randomized	Concealed	12 mos.
Diamond et al. (2010), US	66	12–17	83.3	Patients with suicide thoughts and moderate depression from primary care and emergency rooms	Attachment-based Family Therapy (ABFT)	Enhanced Usual Care (EUC)	Subjects randomized	Concealed	24 weeks
Esposito-Smythers et al. (2011), US	40	13–17	66.7	Suicide attempters in inpatient unit, or with suicidal ideation, and with alcohol or cannabis use disorder	Integrated outpatient cognitive behavioral therapy (I-CBT)	Enhanced Treatment As Usual (E-TAU)	Subjects completed	Concealed	18 mos.
Asarnow et al. (2011), US	181	10–18	69.1	Suicide attempters in ED, or with suicidal ideation	Family intervention for suicide prevention (FISP)	TAU	Subjects completed	Concealed	~2 mos.
Ougrin et al. (2011), UK	70	12–18	80.0	Adolescents referred for assessment for self-harm	Therapeutic intervention (TA)	AAU	Subjects randomized	Concealed	3 mos.
Green et al. (2011), UK	366	12–17	88.5	Self-harm repeaters in CAMHS service	Developmental group psychotherapy	TAU	Subjects completed	Concealed	12 mos.
Mehlum et al. (2014), Norway	77	12–18	88.3	Self-harm repeaters in CAMHS who meet some criteria for DSM-IV BPD	Dialectical behavior therapy (DBT-A)	Enhanced usual care (EUC)	Subjects randomized	Concealed	4 mos.

*BPD, borderline personality disorder; SPT, specific psychological treatment; TAU, treatment as usual; AAU, Assessment as usual; ED, emergency department; ITT, intention to treat*.

Allocation concealment was adequate in ten of the studies (Harrington et al., 1998; Wood et al., 2001; Chanen et al., 2008; Hazell et al., 2009; Diamond et al., 2010; Asarnow et al., 2011; Esposito-Smythers et al., 2011; Green et al., 2011; Ougrin et al., 2011; Mehlum et al., 2014), while it was unclear in two of the studies (Spirito et al., 2002; Donaldson et al., 2005). The Jadad scores were 3 in eight of the studies (Wood et al., 2001; Chanen et al., 2008; Hazell et al., 2009; Asarnow et al., 2011; Esposito-Smythers et al., 2011; Green et al., 2011; Ougrin et al., 2011; Mehlum et al., 2014), 2 in two studies (Harrington et al., 1998; Diamond et al., 2010) and 1 in two studies (Spirito et al., 2002; Donaldson et al., 2005). Disagreements were resolved by a consensus meeting between two of the authors.

Different types of SPT were used in the studies, including: family-based cognitive-behavioral therapy to increase motivation for engagement and care linkage telephone contacts (Asarnow et al., 2011); problem-solving intervention designed to increase adherence to outpatient treatment (Spirito et al., 2002); attachment-based family therapy targeting family processes associated with depression and suicide (Diamond et al., 2010); cognitive analytic therapy (CAT) as early intervention for complex and relational disorders especially borderline personality disorder (Chanen et al., 2008); developmental group psychotherapy incorporating techniques from cognitive behavioral therapy, dialectical behavior therapy, and group psychotherapy (Wood et al., 2001; Hazell et al., 2009; Green et al., 2011); modified dialectical behavior therapy (DBT-A) for self-harm adolescents with borderline personality traits (Mehlum et al., 2014); home-based family intervention by child psychiatric social workers (Harrington et al., 1998); individualized cognitive-behavioral skill-based treatment (SBT) targeting problem solving and affect management skills in adolescents who attempt suicide (Donaldson et al., 2005); integrated outpatient cognitive behavioral intervention to remediate maladaptive cognitions and behaviors in adolescents with co-occurring alcohol or other drug use disorder and suicidality (Esposito-Smythers et al., 2011); and therapeutic assessment, a brief intervention based on CAT, on identifying target problem, enhancing motivation and exploring potential ways to change (Ougrin et al., 2011). These interventions were compared to a variety of control treatments, including TAU, enhanced TAU, assessment as usual and supportive relationship treatment. These control treatments will be referred as “treatment as usual” (TAU).

### Effects of SPT vs. TAU on Treatment Engagement

**Figure 2:**
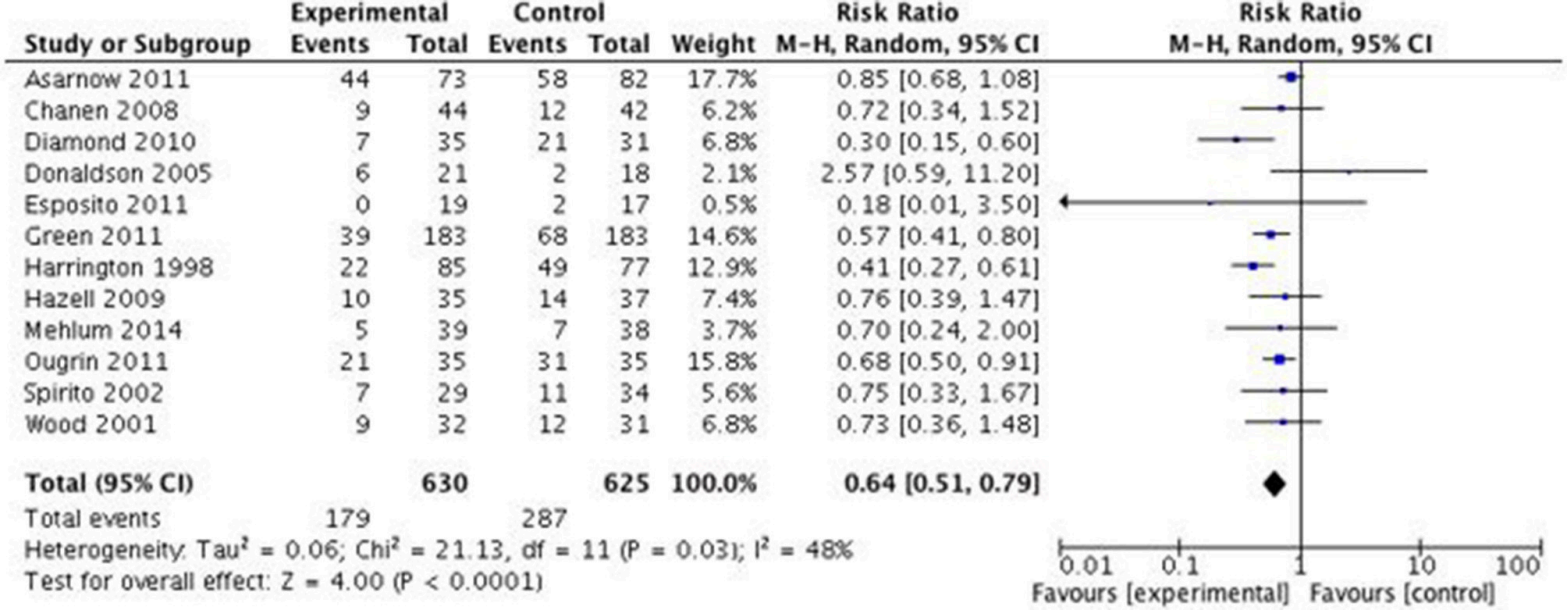
Effects of specific psychological treatment (SPT) vs. treatment as usual (TAU) on treatment engagement in self-harming adolescents. M-H = Mantel-Haenszel.

A full summary of participants' flow and engagement is presented in Table 2. Treatment engagement with SPT and TAU was compared in the 12 included studies (*n* = 1,255). SPT vs. TAU was associated with statistically significant improvement in engagement. The number of subjects not completing four or more sessions is statistically significant between SPT (28.4%, 179/630) than TAU (45.9%, 287/625), RR = 0.64 (95% CI:0.51–0.79, *p* < 0.0001). A significant heterogeneity was found amongst the studies (*I*^2^ = 48%, *p* = 0.03). Complete table of data analysis is presented in Figure 2.

**Table 2:** Participants' flow and treatment engagement reporting effect of SPT vs. TAU.

**Study**	**Eligible**	**Randomized**		**Completed Follow-up**	**Attended 4 or more sessions**		**Mean total N of sessions attended**		**Allocation concealment Q**
		**SPT**	**TAU**		**SPT**	**TAU**	**SPT**	**TAU**	
Harrington et al. (1998)	288	85	77	149	63	28	7.6	3.6	1
Wood et al. (2001)	83	32	31	62	23	19	11.5^*^	4^*^	1
Spirito et al. (2002)	82	36	40	63	22	23	7.7	6.4	2
Donaldson et al. (2005)	44	21	18	31	15	16	9.7	9.5	2
Chanen et al. (2008)	106	44	42	78	35	30	13^*^	11^*^	1
Hazell et al. (2009)	133	35	37	68	25	23	8.8	Not reported	1
Diamond et al. (2010)	69	35	31	57	28	10	9.71	2.87	1
Asarnow et al. (2011)	254	89	92	135	29	24	5.3	3.1	1
Esposito-Smythers et al. (2011)	69	20	20	36	19	15	45.7	24.6	1
Ougrin et al. (2011)	73	35	35	60	14	4	2^*^	0^*^	1
Green et al. (2011)	366	183	183	359	144	115	8.5	9.7	1
Mehlum et al. (2014)	77	39	38	77	34	31	30.9	21.3	1

* *Median was reported*.

**Figure 3:**
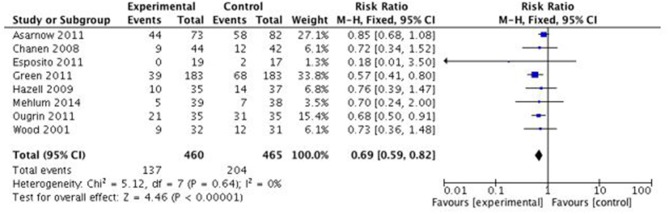
Effects of specific psychological treatment (SPT) vs. treatment as usual (TAU) on treatment engagement in self-harming adolescents (Studies with Jaded score >2). M-H = Mantel-Haenszel.

Four studies with Jadad scores ≤2 were removed in order to perform a sensitivity analysis using fixed effects model. The overall effect in the remaining eight studies remained robust (*p* < 0.00001) in the number of subjects not completing four or more sessions between SPT (29.3%, 137/460) than TAU (43.9%, 204/465), RR = 0.69 (95% CI:0.59–0.82). Complete table of data is presented in Figure 3.

### Meta-Regression

Meta-regression was performed to assess the influence of the number of training sessions (single vs. multiple), year of study, mean age and gender on the effect size. None of the study characteristic variables showed a significant moderating effect on treatment engagement. For trials with single training session, the pooled effect in the number of subjects not completing four or more sessions between SPT (52.5%, 72/137) and TAU (66.2%, 100/150) was not statistically significant, RR = 0.78 (95% CI:0.65–0.93, *p* = 0.486). For trials with multiple training session, the pooled effect in the number of subjects not completing four or more sessions between SPT (21.7%, 107/493) and TAU (39.5%, 187/474) was also not statistically significant, RR = 0.57 (95% CI:0.43–0.75, *p* = 0.145). The RR for not completing four or more sessions was therefore slightly lower in the multiple session trial when compared to the single training session group. The difference in relative risk was –0.32 (95% CI: −0.69–0.049, *t* = −1.86, *p* = 0.089). The number of sessions did not have a significant moderating effect on outcome. For the year of study, the mean difference in relative risk was 0.0191 (95% CI: −0.023–0.061, *t* = 0.14, *p* = 0.371). The mean difference in relative risk for percentage of female was −0.0204 (95%CI: −0.0426–0.0017, *t* = −2.00, *p* = 0.0706). Finally, the mean difference in relative risk for mean age was 0.0198 (95% CI: −0.431–0.471, *t* = 0.088, *p* = 0.931).

### Funnel Plots and Risk of Bias

**Figure 4:**
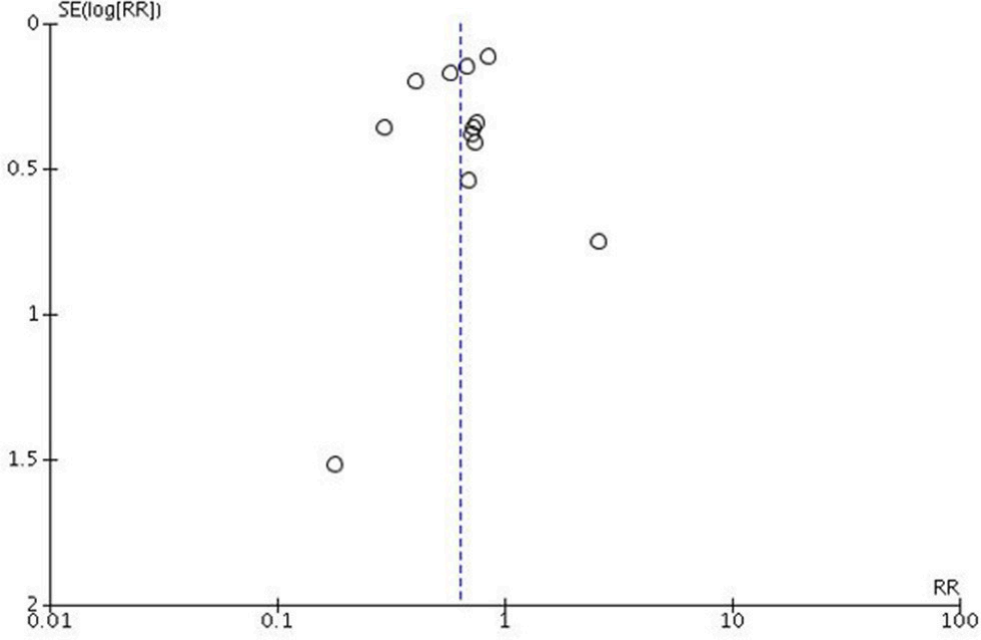
Funnel plot.

There was little evidence of funnel plot asymmetry in this meta-analysis, suggesting that there is no significant publication bias. The funnel plot is presented in Figure 4. The results of Egger's tests indicate that there was no publication bias (*p* = 0.64).

## Discussion

In this meta-analysis, results have shown evidence that SPT leads to better treatment engagement than TAU. Along with the results on efficacy in a recent meta-analysis (Ougrin et al., 2015), the results of the present study support the value of SPT in the management of self-harm. To our knowledge this is the first meta-analysis to demonstrate that offering SPT not only reduces self-harm in adolescents but also increases engagement with treatment. The results of this meta-analysis are different from a previous meta-analysis (Ougrin and Latif, 2011) which failed to demonstrate differential engagement between SPT and TAU. One possible explanation is that the first meta-analysis lacked power to demonstrate this differential effect. Despite this, ~30% of adolescents fail to engage with SPT indicating significant challenges for future research.

Meta-regression has revealed that none of the moderators have a significant moderating effect on treatment engagement, although a weak effect is seen for number of training sessions (single vs. multiple) and the percentage of female. There is a lower risk for multiple training sessions than in single training sessions in SPT compared to TAU. This may imply the importance of having a multiple session treatment although further research is needed, particularly on establishing a minimum of number of sessions required. Furthermore, studies with a higher proportion of female adolescents seem to have a lower risk for not attending four or more sessions, which may imply that female adolescents may have better treatment engagement in SPT compared to TAU. There has been little research done regarding gender differences in treatment engagement. Generally, more females than males seek help for mental health problems while the compliance of male patients is poorer than that of females in actual clinical settings (Hawton, 2000). Similar gender differences have been found in treatment programs for substance abusers, where female programs have significantly higher scores in counselor rapport and treatment participation, with gender being a significant moderator (Staton-Tindall et al., 2007). It is also often recommended to provide multi-session training sessions for adolescents who self-harm. According to NICE guidelines, 3 to 12 sessions should be offered to people who self-harm (National Institute for Health Care Excellence, 2011). Further research on the effects of gender differences and number of training sessions on patients' responses and treatment engagement is needed.

In considering the results of this meta-analysis, several limitations merit note. First, insufficient good-quality, independently replicated RCTs have been conducted to draw firm conclusions about the effectiveness of specific SPTs for engagement in adolescents with self-harm. Small number of RCTs with small number of participants precluded subgroup analyses. There may be underlying differences between the SPT provided by the research team and the TAU delivered in another clinical setting. The research team may have greater motivation than other health professionals in another clinical setting to keep the young people engaged in treatment. Furthermore, therapists from the research team and clinical setting may have very different training and supervision, as the research clinical staff is typically specifically trained for the treatment and under more rigorous supervision, particularly as that clinical staff rarely have much supervision time with their high caseload. These differences could be minimized by providing some training sessions for the clinical staff (Asarnow et al., 2011). Having the research team directly providing the TAU would also be a great way to address these limitations (Chanen et al., 2008).

Regardless of the treatment delivery methods, the results were significant across all but one study. In addition, there were significant differences between the SPTs included in the meta-analysis. There were three different RCTs implementing developmental group therapy to adolescents and significant effect on treatment engagement was seen across those studies (Wood et al., 2001; Hazell et al., 2009; Green et al., 2011). Chanen and colleagues utilized individual therapy without incorporating any parental involvement or group therapy elements (Chanen et al., 2008). In most of the studies, parents were either invited or required to participate in at least a portion of the therapy. Family involvement may influence treatment engagement especially in younger adolescents (Harrington et al., 1998; Spirito et al., 2002; Diamond et al., 2010; Asarnow et al., 2011; Esposito-Smythers et al., 2011; Ougrin et al., 2011; Mehlum et al., 2014). Moreover, previous research has shown that self-harm is often precipitated by family relationship conflicts, which may indicate the importance of involving parents to encourage better communication and resolve any presenting conflicts (Wagner, 1997; Brent et al., 2009). Home-based intervention showed a large positive effect. Although not easily implemented, home visits may be incorporated into a treatment package to enhance engagement (Harrington et al., 1998). Although parental involvement and home visits may be important, not enough replicated RCTs are available to draw firm conclusions about the role of specific components of SPT in maximizing treatment engagement. Furthermore, modern technology has become a dominant gateway of communication, especially in adolescents. Under appropriate designs and consideration, technology-based therapy with the use of internet, social media, and mobile devices may still incorporate elements of traditional therapy such as allowing for family involvement (Cox and Hetrick, 2017). Such therapy may be more appealing to adolescents and could perhaps enhance their engagement. Internet-based raining in psychological therapies also offers promise (Rakovshik et al., 2013, 2016).

The age range of most of the studies was between 12 and 18 years old, with only one study including adolescents as young as 10 years old. Although self-harm is most common in adolescents and young adults, first self-harm episode is reported by the age of 12 years in a third of patients with borderline personality disorder (Zanarini et al., 2006). It may therefore be particularly important to include younger participants in the future studies.

Studies with interventions that did not require young people to attend treatment sessions were excluded in this meta-analysis. In our search, this has excluded the studies where the participants were the parents, instead of the young people themselves, in order to measure treatment engagement of young people directly. There is emerging evidence that electronic therapy without face-to-face element may have some benefits on young people. We did not identify any RCTs that investigated the effects of electronic therapy on self-harm. Furthermore, patients seldom have the choice of receiving the treatment of their choice and it may impede the likelihood of sustained engagement. While it is impossible to allow for actual choices in RCTs, it is somewhat equally difficult to provide treatment choices clinically as it is often limited by resources and of best inters to the patients. However, arrangement over the mode and delivery of treatment may be plausible. An investigation on the preference over various means of treatment may identify crucial indication of specific elements in treatment for self-harming adolescents. Future research could aim on incorporating elements of electronic therapy into traditional face-to-face therapy in order to investigate its effect on treatment engagement, as the electronic therapy could potentially be a bridge in between the traditional therapy sessions.

Treatment engagement was defined as attending four or more sessions in this meta-analysis. This cut-off has been used in a lot of research studies (Spirito et al., 1992; Wood et al., 2001). Alternative cut-off points were considered, including the total number of sessions and attending at least one follow-up session. The former provides a good overall picture of treatment engagement, but it could be prejudicial for those who do not attend many sessions due to the resolution of their symptoms or for those who do not feel they benefit from the treatment. The latter is an easily replicable and available measurement, but it does not acknowledge the dose-response relationship. Adolescents may not be required to attend treatment sessions in some clinical settings nowadays, however their treatment engagement remains questionable under such circumstances. Future studies could look at alternative ways of measuring treatment engagement.

Although all SPTs were compared against routine care, some studies enhanced or designed a specific TAU, which may be significantly different than usual routine care (Donaldson et al., 2005; Esposito-Smythers et al., 2011). As more RCTs become available in the future, more subgroup analyses could be performed to see whether such differences in the TAU arm have an influence on the effect. All studies had primary outcome measuring something other than engagement, except for two studies (Spirito et al., 2002; Ougrin et al., 2011), which implies significant heterogeneity in study design. In addition, there were significant differences in self-harm definitions used by different studies, which further complicate interpretation of the results. This is due to a lack of consensus in the definition of self-harm. Recently, non-suicidal self-injury (NSSI) has become more prevalent and was proposed in DSM-5 that it should become a distinct diagnostic category (American Psychiatric Association, 2013). A lot of young people have NSSI as their main problem but are not currently diagnosed with such. Hence, there are often discrepancies and difficulties when psychotherapies are being delivered. The proposed DSM-5 diagnostic criteria for an NSSI disorder include a criterion that the behavior causes the person impairment or distress and there is a discussion on whether this criterion should be part of each diagnosis. It has been found that adolescents with NSSI without impairment or distress did not fulfill criteria for borderline personality disorder and had less externalizing disorders (In-Albon et al., 2013). Future research on treatment engagement and effectiveness could also look at differences between such populations of young people. As we move to a better consensus in the definition of self-harm, SPT for self-harm could be better researched, designed and implemented.

## Conclusion

Treatment engagement is crucial in psychological therapy. Engaging adolescents with psychological treatment is necessary although not sufficient to achieve treatment goals Specific psychological treatments should be offered to adolescents with self-harm to maximize treatment engagement. More research is required to delineate specific characteristics of SPT linked with better engagement. Greater international consensus regarding definition of self-harm should facilitate research in this field.

## Author Contributions

SY is responsible as the corresponding author. She completed literature search and was primarily responsible for writing the content of this article. KK completed literature search and rating of each articles. He attended all consensus meetings. He helped to review and edit the article. DO provides approval for publication of the content. He supervised and led SY and KK throughout the process of writing this article. He helped to review and edit the article.

### Conflict of Interest Statement

The authors declare that the research was conducted in the absence of any commercial or financial relationships that could be construed as a potential conflict of interest.
